# Reading the World through the Skin and Ears: A New Perspective on Sensory Substitution

**DOI:** 10.3389/fpsyg.2012.00457

**Published:** 2012-11-07

**Authors:** Ophelia Deroy, Malika Auvray

**Affiliations:** ^1^Center for the Study of the Senses, Institute of Philosophy, University of LondonLondon, UK; ^2^Laboratoire d’Informatique pour la Mecanique et les Sciences de l’Ingenieur, UPR 3251, CNRSParis, France

**Keywords:** sensory substitution, perception, plasticity, reading, dual-route models

## Abstract

Sensory substitution devices aim at replacing or assisting one or several functions of a deficient sensory modality by means of another sensory modality. Despite the numerous studies and research programs devoted to their development and integration, sensory substitution devices have failed to live up to their goal of allowing one to “see with the skin” (White et al., 1970) or to “see with the brain” (Bach-y-Rita et al., 2003). These somewhat peremptory claims, as well as the research conducted so far, are based on an implicit perceptual paradigm. Such perceptual assumption accepts the equivalence between using a sensory substitution device and perceiving through a particular sensory modality. Our aim is to provide an alternative model, which defines sensory substitution as being closer to culturally implemented cognitive extensions of existing perceptual skills such as reading. In this article, we will show why the analogy with reading provides a better explanation of the actual findings, that is, both of the positive results achieved and of the limitations noticed across the field of research on sensory substitution. The parallel with the most recent two-route and interactive models of reading (e.g., Dehaene et al., 2005) generates a radically new way of approaching these results, by stressing the dependence of integration on the existing perceptual-semantic route. In addition, the present perspective enables us to generate innovative research questions and specific predictions which set the stage for future work.

## Introduction

Since the 1960s, a series of devices have been developed to replace or assist one or several functions of a deficient sensory modality (e.g., vision) by means of another sensory modality (e.g., touch or audition). These devices have been primarily developed to help sensorially impaired people to navigate in their environment and to recognize objects. Contrary to white canes which only rely on the relevant initial properties of an object (in this case, mechanical), these devices are based on a technologically guided conversion of a certain type of stimuli, the perception of which is deficient, into another type of stimuli, for which receptors are intact (Bach-y-Rita et al., 1969). The first and probably most well known of these sensory substitution devices has been designed to compensate for visual deficits and convert visual images obtained through a camera into patterns of tactile stimuli. The tactile-visual sensory substitution (TVSS) designed by Bach-y-Rita in the 1960s was the first device to launch the optimistic claim that blind individuals could “*see* with the skin” (White et al., 1970, emphase are our own). The very idea of sensory substitution has been extended to the conversion of visual images into auditory signals and led to the development of devices like the vOICe (for “Oh I see,” see Meijer, 1992), the prosthesis for substitution of vision by audition (PVSA, see Capelle et al., 1998), or the Vibe (Hanneton et al., 2010). The pressing challenges faced by researchers consist in finding the best way to provide blind users with more and more accurate information usually allowed by vision, such as color, shape, or distance. A more fundamental question, though, is to understand what underlies the acquisition of new identification skills that are usually characteristic of a certain sensory modality by means of another.

The initial idea of sensory substitution and the research conducted since have encouraged the idea that trained users of visual-to-tactile or visual-to-auditory conversion systems such as the Tongue Display Unit, the vOICe, or the PVSA, recover a form of vision – or at the very least, come close to acquire an analog to sensory perception. Understood in a superficial way, such claims need not be challenged: the capacity to respond in a discriminative way to a certain kind of stimuli (or changes thereof) can qualify as perceptual (see Garner et al., 1956) and the devices advertised as sensory substitution certainly satisfy this requirement. Robust evidence shows that sensory substitution devices provide their users with new abilities to detect and/or respond to changes in their environments. However, this repeated, but hardly ever examined equivalence between using a sensory substitution device and perceiving through a canonical sensory modality has turned into the dominant framework in which to interpret the data obtained with those devices. Implications of sensory substitution for understanding brain plasticity (Amedi et al., 2007; Bubic et al., 2010; Ortiz et al., 2011), sensory individuation (Hurley and Noë, 2003; Auvray and Myin, 2009; O’Regan, 2011), or even synesthetic unions (Proulx and Stoerig, 2006; Proulx, 2010; Ward and Meijer, 2010) are all drawn with this assumption in mind, to the risk of generating a confirmation bias in favor of the perceptual nature of sensory substitution. But is another understanding of sensory substitution possible, or even recommended?

Despite the numerous studies and research programs devoted to their development and understanding (see Auvray and Myin, 2009, for a review), it is important to underscore that sensory substitution devices have failed to live up to their goal of offering something close to the speed, accuracy, or discriminatory responses observed in non-impaired individuals. Sensory substitution devices are even further away from allowing their trained users to see with the skin or ears: they do not offer something somewhat similar to the rich visual experience that sighted people have. But where do these limitations come from? Contrary to the optimistic, but ungrounded idea that limitations result solely from technological constraints and could be overcome in the near future, we want to suggest that they might be intrinsic to sensory substitution, and suggest that it will never come close to what perception can be in other “natural” senses. The intrinsic limitations, in our sense, are not restricted to a difference in the accuracy of tactile or auditory processing that would be insufficient to code for visual objects. Beyond these sensory differences, a fundamental problem comes from the learning of a new sensory translation code. It is only by a better understanding of what learning to use sensory substitution devices amounts to that we can understand why they are not similar to perceiving in a typical modality.

The fact that a certain amount of familiarization and training is necessary before being able to obtain basic skills with a sensory substitution device (e.g., Kaczmarek and Haase, 2003; Auvray et al., 2007a) could been seen as a straightforward objection to the equivalence between sensory substitution and classical perception. As we will discuss further below, traditional senses are generally taken to be operational without training. However, what seems to us most problematic for the perceptual equivalence relies less on the necessity of training *per se* than on what training amounts to. As we will detail in Section “Fifty Years of Evidence under the Perceptual Assumption,” the past 50 years of research on training and integration of sensory substitution devices has highlighted some positive, but limited, evidence regarding the results and the nature of training, as well as regarding the changes in subjective experience and neural organization that follows from longer-term use. Despite being difficult to interpret, this set of evidence has been increasingly considered as a confirmation of what we describe as a perceptual assumption. It generates a confirmation bias as both the experimental designs and the interpretations of the results are oriented to reinforce the perceptual assumption. As argued at the end of Section “Fifty Years of Evidence under the Perceptual Assumption,” the perceptual interpretation of sensory substitution has consequences for its theoretical and technological investigation, all of which might benefit from its revision.

In Section “A New Model Based on the Analogy with Reading,” we propose an alternative understanding of sensory substitution based on an analogy with the acquisition of reading skills. Like reading, using sensory substitution devices requires training and results in the progressive automatic decoding of meaningful information. More specifically, in these two cases, the information which was previously available and structured through one sensory modality is artificially made available through another sensory modality thanks to a code purposively designed to preserve the relevant structure and dimensions. The aim is therefore to ensure recognition of the same objects across a change of medium. Once understood with this goal in mind, it is easier to consider the skills obtained through sensory substitution as *crafted onto* some existing route(s) which go from sensory stimulation through to recognition. Instead of opening an autonomous route to previously inaccessible information, sensory substitution needs to be conceived as being a derived route which requires that a first route and ways of accessing information already exist. The analogy with dual-route models of reading then provides a novel way to specify this proposal and understand the integration of sensory substitution devices. More importantly, besides offering a novel perspective on the neurological, experiential and behavioral effects of sensory substitution, the dual-route analogy also generates novel predictions about training, and suggests new directions for neurological investigation, as highlighted in Section “Conclusion.”

## Fifty Years of Evidence Under the Perceptual Assumption

### Scope of the assumption

The various data about training, subjective changes and brain plasticity that underlie the acquisition of new identification and localization abilities have been unanimously interpreted through a perceptual assumption. That is, as showing that using sensory substitution devices is identical – if not in practice, at least in principle – with the exercise of canonical sensory modalities. Starting with White et al.’s (1970) claim that users of visual-to-tactile substitution devices would be “seeing with the skin” to Bach-y-Rita et al.’s (2003) revised idea that they would be “seeing with the brain,” these optimistic claims have been echoed to the wider audience including potential users of these devices: sensory substitution devices have been advertised as “rewiring brains to see with sound” and “restoring a form of sight to the blind” (Trivedi, 2010, p. 42). The idea that sensory substitution devices could be analogous to the visual system also finds an illustration in the domain of technology, with the development of the PVSA, where a higher resolution has been given to the center of the picture to replicate what occurs in the fovea (Capelle et al., 1998).

Claims that technological supplementation could allow the user to regain something similar to what perception normally is have also been embraced in the discussion of the philosophical implications of sensory substitution. Many are keen to accept Heil’s idea that “a person making intelligent use of a TVSS may be said to be seeing (though perhaps only dimly) features of his environment” (Heil, 1983, p. 16). Opposing these visual claims has mostly meant objecting that using a device like the Tactile Vision Sensory Substitution is more properly described as remaining as a form of tactile perception (see Block, 2003).

**Figure 1:**
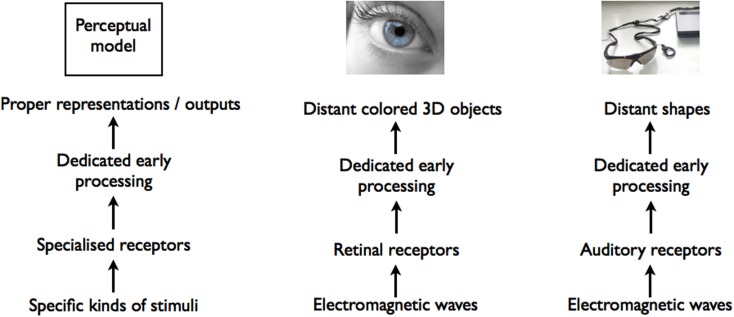
**The analogy between reading and integrating visual-to-auditory sensory substitution devices (like the vOICe)**. Full arrows indicate new elements brought about by training and new devices or artifact (coded letters in the case of reading; decoding device in the case of sensory substitution devices), dotted arrows indicate elements that pre-existed.

These debates remain based on an implicit perceptual equivalence which we suggest to identify as a perceptual assumption. The influence of this perceptual assumption is visible in the fact that researchers accept or target equivalences between using a sensory substitution device and the exercise of a sensory modality. In other words the perceptual assumption considers that sensory substitution follows what occurs with canonical cases of perception through one of the typical sensory modalities, that is as specialized channels for transducing external information. As spelled out by Grice (1962), perceiving through each of these specialized sensory routes typically starts out with specific kinds of receptors being stimulated by certain kinds of stimuli; the information is then further processed (at least at an early stage) by dedicated sensory mechanisms that finally deliver a representation of a certain kind of object or properties or leads to specific responses (see Figure 1). Even though perception of certain objects can be multisensory, it is considered as constituted of two or more converging channels, each of which can be specified independently through the four criteria of stimuli, receptors, processes and outputs. When understood as perceptual in this sense, sensory substitution is also seen as fitting with these criteria. This explains the two main perspectives that are currently present in the literature: some consider sensory substitution devices from the point of view where they lead to outputs that resemble those produced by vision, or where they respond to visual stimuli, and think of it as sight or substitute for it; others consider them from the point of view where they recruit the receptors and early stages of processing of an existing sense, and take it to be a sensory reorientation or extension of the existing sense they exploit, for instance touch or audition. In both cases, though, sensory substitution is forced into this single route model used for other sensory channels.

The problem with the perceptual assumption is not only that it delivers an unstable verdict regarding the exact sensory modality to which a certain device should be ascribed. A more fundamental problem than to decide whether using a device is closer to seeing or hearing, is to examine whether the theorizing concerning these devices should remain bound to the perceptual framework where, canonically, perceiving means channeling information through a single, dedicated route.

To us, the perceptual assumption appears to have led to a confirmation bias in the interpretation of the results. The results of existing studies have been systematically filtered out of the negative evidence, or data fitting less well with this assumption, while the remaining evidence has been seen as confirming the equivalence between using a sensory substitution device and perceiving through one of the canonical senses. What’s more, the experimental protocols themselves are built with the perceptual assumption in mind which, in turn, constrains or limits the kind of data that can be gathered. What we want to stress here is the tension between the perceptual model and parts of the data previously mentioned.

### A confirmation bias regarding performance and training

What initiated and sustained the research interest for sensory substitution in the first place is the fact that users of encoding and decoding devices can perform tasks that they would not be otherwise able to do given the general or temporary impairment of one of their senses. In the case of visual-to-tactile devices, most users are, within an hour or two, able to walk around and navigate in their environment, and avoid obstacles. They also start to locate objects in space (Jansson, 1983) and describe their shapes (Sampaio et al., 2001; Kaczmarek and Haase, 2003). Ease and performance improve with practice: the estimated amount of training needed to reach a reasonable level of performance with the Tongue Display Unit is approximately 8 h (Kaczmarek and Haase, 2003). One interesting result obtained with these devices is that users also become able to make perceptual judgments using perspective, such as the increasing angular size of approaching objects (White et al., 1970; Bach-y-Rita, 1972); a somewhat surprising result given the bi-dimensional nature of the stimulation. Another interesting phenomenon, investigated under the name of “distal attribution,” suggests a change in users’ subjective experience: they no longer report feeling the stimulation on their skin, where it occurs, but directly attribute the cause of the stimulation to a distant object (Bach-y-Rita et al., 1969; White et al., 1970; Bach-y-Rita and Kercel, 2003). This said, this much quoted aspect of experience with visual-to-tactile sensory substitution device comes mostly from subjective reports and awaits further psychophysical testing.

Similar results have been obtained with visual-to-auditory devices such as the vOICe and the PVSA. Trained users are able to move around and to point at objects in their; to recognize and categorize various objects (see Arno et al., 2001a,b; Renier et al., 2005a; Auvray et al., 2007a; Proulx et al., 2008).What is striking with these two visual-to-auditory conversion systems is that users can reach a proficient level of performance in a rather short period of time, in the absence of a straightforward analogical code: in other words, in the absence of a direct stimulus equivalence as exists, for instance, between visual shapes and tactile shapes. The fast integration of the previously described visual-to-tactile devices can by contrast be attributed to the fact that they exploit pre-existing or partly hard-wired crossmodal equivalence between visual shapes and tactile shapes (see Streri, 2012, for a recent review). This means that these crossmodally equivalent stimuli are easily treated as conveying information about the very same property. The translation code which consists of going from the tactually sensed shapes caused by the device to the visual shapes initially encoded is in this sense already known to sighted and late-blind users, and probably benefits from its being hardwired in congenially blind users, as suggested by the fast acquisition of visuo-tactile equivalence in newly sighted individuals. If individuals whose sight was restored are able to visually match an object to a haptically sensed sample, this transfer is not immediate but develops rapidly (see Held et al., 2011). Visual-to-auditory devices, by contrast, make use of a largely arbitrary code. In the vOICe, for instance, the scene is scanned from left to right, and higher frequenies codes for higher positions in the field, while loudness codes for brightness^1^.

For visual-to-auditory devices, at least, learning or familiarization thus appears important. The main question here concerns the kind of learning that is taking place. Although many users come to wear the device knowing its function or being explicitly told what the translation codes are, it is still not clear which strategy is then used or how important or necessary sensorimotor feedback is to learning (e.g., Auvray et al., 2005; Siegle and Warren, 2010). The first thing to underscore here is a bias in calling the performance perceptual. The skills gradually acquired by users are always presented with respect to the perceptual framework: the distal attribution reported with visual-to-tactile devices has been understood as a switch from sensation in the substituting modality to a new perception of an external object (Bach-y-Rita and Kercel, 2003); The new localization abilities are said to come within a “new perceptual space” (Auvray et al., 2005, p. 520). Last but not least, the new identification skills acquired by the users are interpreted as them gaining access to new perceptual qualities, for instance, accessing shapes through audition (e.g., Heil, 1983).

The level of performance achieved through any of these sensory substitution devices remains however inferior to any of the perceptual standards we know of. If one considers the standards of the intact stimulated modality (audition or touch), sensory substitution is lower in terms of speed and accuracy, and lower in terms of automaticity and effort. The same is true if the chosen standards are borrowed from the modality which is supposed to be compensated. Noticeably, compared to visual perception, the number of objects which can be jointly accessed or available through sensory substitution devices seems to be much lower. Contrary to vision, where people have been shown to be able to track multiple objects (e.g., Pylyshyn, 2004), users of sensory substitution devices have not been shown to be able to track or identify multiple objects at once. Thinking then in terms of resolution, the same limits apply: visual-to-tactile sensory substitution devices might never reach the maximal resolution obtained with vision. According to Loomis (2010; see also Loomis and Klatzky, 2007), each sensory modality can be characterized by a certain spatial bandwidth which corresponds to the total capacity for conveying spatial information. The visual one is unique in allowing a wide field of view and an access to fine spatial details. By contrast, tactile processing has a lower spatial bandwidth (e.g., Loomis, 1981). Such a large difference in spatial resolution between the two senses involved means that it might be impossible for touch to ever substitute for vision in complex real-world situations, such as driving a car or playing basketball.

Thinking that the use of a sensory substitution device comes close to perceptual experience also misses the fact that certain elements typically associated to sensory experiences are lacking. Noticeably, the shapes perceived in one sensory modality are not directly associated to pleasures or pains felt while perceiving the same shape in another sensory modality (Lenay et al., 2003). It should, however, be mentioned here that there are similar reports of the absence of emotion and meaning felt by persons blind from birth who recover sight following the removal of cataracts. There are, at least at the beginning, no affective qualities associated to colors and seeing faces is not associated to any emotional content (Gregory, 2003).

What about training? The very necessity of training, which has been in the background of the studies investigating performance, is also forced into the perceptual framework, and this despite an apparent tension with the common-sense notion of perception (see Deroy and Auvray, in press). Although various perceptual systems are considered to develop through time in infancy (Johnson and Vecera, 1996; see also Bremner et al., 2012, for a recent take on this issue), this is not widely taken to require training; and especially an explicit verbal training such as the one usually given to obtain good results with sensory substitution devices. Interestingly though, the necessity of training has not been seen as a counter-argument to the perceptual model. Some have taken it as a sign that the use of sensory substitution devices could be seen as analogous to the acquisition of perceptual expertise through an existing modality (see Heil, 2011). Others, stressing the role played by active exploration during training with sensory substitution devices, suggest that their progressive integration actually confirms sensorimotor views of perception where sensory perception comes with the learning of new sensorimotor contingencies (O’Regan and Noë, 2001; O’Regan, 2011). What matters here is not to adjudicate between these two versions, but to underscore that each one manages to square the necessity of training with the perceptual model.

Looking more closely at the contents of training though might be important. Although active exploration is useful or even constitutive, the identification of objects through sensory substitution devices also integrates conceptual or semantic components which are already present or known. For instance, the identification of a 90° angle through the vOICe is not totally new. The capacity to do so relies on existing knowledge of what a right angle is. Thus, background knowledge does more than building up a form of perceptual expertise in the substituting modality: the acquired expertise is constituted by learning to judge patterns of stimulation *as* falling under a certain concept or category. What happens is that the judgments become progressively faster, more accurate and more automatic. On important thing to underscore here is that pre-existing cognitive elements play a direct role in the integration of sensory substitution devices: users identify novel objects more rapidly when they belong to the same category as the objects that were used during training (e.g., see Kim and Zatorre, 2008) and they are slower for objects belonging to categories that were not used during training. Patterns of generalization suggest that semantic knowledge about objects categories, as much as actual physical similarities perceived through the sensory substitution device, plays an important role in enabling successful performance.

#### Interim summary

The perceptual perspective widely taken on sensory substitution devices since their introduction has, overall, simplified the number of terms used to analyze their use and integration. The understanding of sensory substitution has been framed merely in terms of getting information from an intact, pre-existing receptor to the emergence of new responses or representations, analogous to the ones obtained through other known senses. However, several elements contradict the fact that sensory substitution behaves like a classical sensory channeling of information. In terms of results or outputs, sensory substitution is much more limited, for instance, regarding the number and complexity of objects it gives access to than any other form of perception, be it through vision, audition, or touch. The starting point of sensory substitution is also more complex than just having intact receptors and early processing: existing cognitive and semantic components are obviously recruited in the process, and need to figure in the explanation of both the positive and the limited scope of the results. The generalization of training to new objects is also facilitated when these objects remain in the same semantic category (Kim and Zatorre, 2008) and, to our knowledge, users have not been tested for totally arbitrary or novel kinds of stimuli (or even impossible figures) for which they lack pre-existing knowledge.

### A confirmation bias regarding the subjective and neurological changes accompanying training and novel performance

The evidence of novel performance both in localization and identification and the study of training are closely connected. They have led researchers to pay closer attention to further changes accompanying these progressive results, with the double objective of trying to understand what underlies changes in performance and to improve the efficiency of these devices.

The changes in subjective experience are perhaps one of the most striking cases where evidence is forced into a perceptual assumption. As was noted above, users of visual-to-tactile sensory substitution devices do not just learn how to navigate in their environment and to identify distant objects, they also report the feeling of experiencing the distal object. This has been famously described by Guarniero: “very soon after I had learned how to scan, the sensations no longer felt as if they were on my back, and I became less and less aware that vibrating pins were making contact with my skin. By this time objects had come to have a top and a bottom; a right side and a left; but no depth – they existed in an ordered two dimensional space.” (Guarniero, 1974, p. 104). It should be remembered that this specific subjective report came from a philosophy student (not exactly a naïve experimental participant) and thus, we would argue, needs further psychophysical evidential support. Yet, this single statement has played a key role in the discussion of the perceptual status of sensory substitution, at least in philosophy (see Heil, 1983, 2003, p. 228, Heil, 2011, p. 288; Leon, 2011, p. 165; Peacocke, 1983, p. 15, Proulx and Stoerig, 2006). More recent studies have reported a correlation between the reports of new phenomenological experiences of flashes in trained late-blind users of visual-to-tactile sensory substitution devices and the recruitment of the occipital cortex (Ortiz et al., 2011), but such changes are not documented in congenitally blind individuals. The visual character of these induced flashes or lights thus remains to be ascertained in the case of individuals with no residual vision.

Subjective changes have also been documented with visual-to-auditory devices. Based on two subjective reports in late-blind users, Ward and Meijer (2010) have suggested that training with the vOICe could lead to the occurrence of visual images, comparable to the synesthetic experiences enjoyed by colored-hearing synesthetes (see also Proulx, 2010, for an analogous claim). However, the evidence remains fragile, and compatible with the resurgence of color or texture memories for familiar objects, rather than with the emergence of what Proulx (2010) calls a “synthetic synesthesia.” Looking for subjective changes, and trying to see whether they become visual is strongly oriented by a perceptual assumption, and plays a major role in the question that philosophers have been eager to address, i.e., to what kind of perception the use of sensory substitution device belongs. The nature of the experience is supposed to help determining whether this perception remains in the substituting modality, be it auditory (e.g., with the Voice) or tactile (e.g., with the TVSS, see Humphrey, 1992; Keeley, 2002; Block, 2003; Prinz, 2006) or goes to the substituted modality (e.g., vision for both the vOICe and the TVSS; see Cole, 1990; Dennett, 1991; O’Regan and Noë, 2001; Hurley and Noë, 2003).

Whether we could obtain a determinate answer regarding the phenomenology of trained users of sensory substitution devices is, however, questionable. First, the answer to this question relies heavily on philosophical theories of what “auditory,” “visual,” or other modal signatures are (i.e., what philosophers call kinds of phenomenal character or experiences). Turning, then, to the actual data, the answers provided by users are far from clear. In one of the rare studies dealing with the issue, blindfolded sighted participants were asked to compare the subjective experience of using the visual-to-auditory device the vOICe to other kinds of sensory experiences (Auvray et al., 2007a). For many participants, the modal characteristic varied with the task (being more often visual for localization, and auditory for identification). Some participants even considered the experience to be like an unusual modality – such as having a sonar sense. The results from subjective questionnaires certainly need to be handled with care, especially as the very fact of raising the question can bias participants in trying to guess what the experimenter expects. A minimal conclusion is that the experience of using a sensory substitution device remains endowed with a characteristic feeling and that the experience can feel relevantly different from the experiences that one usually has in the modality stimulated with the device (touch or audition), while making it quite often comparable (but not similar to) previous experiences in the visual modality (see also Kupers et al., 2011).

Besides these subjective changes, more robust data on the use of sensory substitution devices come from the documentation of neurological changes. The most investigated change concerns the increased activation in the visual cortex after practice with visual-to-auditory sensory substitution devices (De Volder et al., 1999; Arno et al., 2001a; Renier et al., 2005b; Collignon et al., 2007) and after practice with visual-to-tactile sensory substitution devices (Ptito and Kupers, 2005; Ptito et al., 2005). The neural plasticity in sensory substitution has been here seen as being of wider interest to the study of neural plasticity following sensory deprivation or impairment (e.g., Collignon et al., 2009).

Some connections between the subjective and neurological changes can be tempting to make, and start to emerge, for instance, with the hypothesis that long-term users of the vOICe could both be subject to neurological reorganization in the occipital cortex (e.g., Ortiz et al., 2011, and have visual experiences of colors or textures (Ward and Meijer, 2010). However, such claims should be taken with caution, given the presence of conflicting evidence obtained with visual-to-tactile devices. In particular, Kupers et al. (2006) used TMS on V1 of blind and sighted participants before and after training with the Tongue Display Unit. Before training, no subjective tactile sensations were reported. After training, some of the blind participants reported tactile sensations that were referred to the tongue (three out of eight early blind and one out of five late-blind participants). The author concluded that stimulation of the visual areas induces tactile but not visual sensations in trained blind users of the Tongue Display Unit. In other words, the recruitment of V1 does not necessarily mean that the associated subjective experience is *visual*. Whatever the answers to this problem, these debates still revolves around comparing using sensory substitution device and perceiving in a canonical sensory modality.

Changes beyond primary sensory areas are now being documented. Amedi et al.’s (2007) study, for instance, revealed that blind and sighted expert users of the vOICe show specific activation in the latero-occipital tactile-visual area (LOtv) when recognizing objects through the shape information conveyed by the vOICe soundscapes. This does not occur when they are merely associating soundscapes to objects or recognizing objects by their typical sounds. The LOtv, which is a locus of multisensory convergence, is usually activated both by the visual and the tactile exploration of objects. Although there are good reasons to see this as a perceptual reorganization, this study suggests that some more complex reorganizations than strictly sensory ones are taking place, having to do with later or supra-modal (in this case, shape) recognition; rather than necessarily with primary sensory processing. Moreover, as reviewed in Bubic et al. (2010) and Ortiz et al. (2011), finer differences might also exist between the late blind, the early blind, and sighted users of sensory substitution devices in the recruitment of occipital areas. Noticeably, it is not yet clear that activation in the occipital cortex reflects genuine bottom-up activation for tactile or auditory stimulation, consistent with what occurs in sensory perception, rather than top-down visual imagery mechanisms (at least in late blind and sighted individuals, see Poirier et al., 2007a,b; Tal and Amedi, 2009).

#### Interim summary

The perceptual assumption dominates the interpretation of the results and orientates most of the research programs. Technical improvements and training are made with this perceptual assumption in mind, leaving the limited success of sensory substitution devices among blind individuals unexplained, or a matter of prophetic improvement. Many scientists postulate that the limited results obtained with sensory substitution devices are only transient and that the gap with sensory perception can be bridged through further technological development or training. As yet, no good arguments have been offered to support this prediction, whereas the robustness of the negative evidence as well as the limited success of the publicly advertised “substitution” devices among blind users speak against the idea that sensory substitution devices will really deliver on this perceptual promise. As we have shown, the perceptual assumption is largely biased and leads one to overlook some important features of the use and integration of sensory substitution device. It has led to premature, and irresolvable debates regarding the analogy with synesthetic rewiring of the senses (Proulx, 2010; Ward and Meijer, 2010), or with visual perception (e.g., Bach-y-Rita and Kercel, 2003; Hurley and Noë, 2003). A better model is needed, that can take all of the evidence into account in a more comprehensive and potentially fruitful way.

## A New Model Based on the Analogy with Reading

The previous review opens an obvious challenge: how can we make sense of the positive evidence collected within the perceptual assumption, while acknowledging the limits and negative evidence that has just been listed? Notice here that a failure to find an alternative model would, indirectly, validate the perceptual assumption as being the best *available* model we have for thinking about sensory substitution. We believe, however, that an alternative can be found, which lifts the apparent contradiction between the canonical and less canonical perceptual aspects of sensory substitution. This alternative is to be found in an analogy with other acquired skills and forms of automatic recognition – namely reading skills.

By analogy, we mean more than the similarity mentioned here or there that converting sounds into visual or tactile signs acts as a precursor to the more recent devices converting images into sounds or tactile stimuli (Bach-y-Rita and Kercel, 2003, p. 541; Eagleman, 2011). The deeper resemblance between the acquisition of reading skills and the integration of sensory substitution devices comes from the fact that, in both cases, the progressive automatization of new identification skills consists in building a second route, which presupposes the existence of a first sensory route *and* parallels it. Instead of being like a dedicated route, reading (typically, visual letters/word recognition) relies on speech perception (classically, auditory speech perception) and delivers access to the very same objects (word meaning). The necessity of a pre-existing sensory route to a specific object, the fact that these properties get *conventionally* re-coded and made available to another sensory modality while not being, or becoming, common between the two is, as we want to stress, what also makes the use and integration of sensory substitution devices sufficiently different from crossmodal transfers such as the one which exists between the visual and tactile identification of shapes (see Streri, 2012) or from other cases of sensory merging (Ernst and Bülthoff, 2004). Thinking about sensory substitution devices by analogy with reading therefore goes away from the perceptual assumption, where the devices resemble a canonical sensory modality, but also from simple claims about the multisensory aspects of these devices.

### Parallels between reading and sensory substitution

Reading is classically defined as learning to access words through vision instead of audition; or eventually, through touch instead of audition in the case of Braille. Interestingly though, it is not straightforwardly a way to gain new visual perceptions – as both shapes and colors/contrast perceived in reading can already be visually perceived. No new receptor is needed to access them. At the same time, strictly speaking, reading brings about something new, which is the possibility to access through vision some objects (words, sentences, and from there meanings) that were only available through audition before. This does not mean that reading adds access to *auditory* objects – as the shapes on the page do not have properties detectable by audition. Reading does not constitute an independent visual road substituting for the auditory perception of spoken language. More importantly, it is not an autonomous or dedicated perceptual system, and it is well explained as the development of *a second route*, grafted onto the auditory speech route, as popularized by the dual-route models of reading (e.g., Coltheart and Rastle, 1994; see also Rastle, 2007 for a review on current models of visual word recognition).

Writing systems have been designed to preserve the phonemic structures that are relevant to access semantic information. In this sense, the code remains “auditorily phonemically” constrained or governed. The acquisition of reading itself relies on existing phonemic skills, not just on auditory perception, and consists in *mapping* what one hears onto what one sees through the mediation of what one knows the later means. It is only as a result of mapping the known written signs to known spoken words and phonemes that readers can progressively entertain auditory representation on the basis of visual words, and this even for unknown or novel items.

Reading, then, is far from merely being a straightforward sensory remapping. Even more specifically, the acquisition of reading is accompanied by a change in subjective experience: written words no longer appear as colored shapes but as meaningful graphemes and words, which get easily and naturally associated to sounds (i.e., either ones that one imaginatively hears, see Spence and Deroy, in press for a discussion; or ones that one should articulate, see Galantucci et al., 2006 for a review). No such documented change in experience, to our knowledge, has been observed as a consequence of the progressive establishment of visuo-tactile crossmodal transfer (see Held et al., 2011). This said, both reading and other crossmodal transfers are internalized and become automatic, in such a way that they no longer require attention or effort from the trained reader.

Finally, learning to read induces crucial neurological changes (noticeably in bilateral dorsal occipital areas associated with higher-level visual processing, in superior temporal areas associated with phonological processing, and in the angular gyri as well as in posterior middle temporal regions associated with semantic processing, see Turkeltaub et al., 2003; Carreiras et al., 2009; Dehaene et al., 2010), probably exploiting human neural plasticity or recycling. New cultural inventions such as reading are closely linked to “the constraints of our brain architecture” (Dehaene, 2009, p. 146) but they are most certainly revealing the extensive, and partly culture-driven, character of brain plasticity (see Ansari, 2012, for a recent review) which other tools and devices certainly exploit.

All these features, we argue, offer an analogy robust enough to think about the results obtained through sensory substitution devices within a reading framework. The relevance of the analogy with reading is more likely to be found with sensory substitution devices that do not use an analogical format (e.g., visual-to-auditory systems such as the vOICe).

### Pushing the analogy further: Dual-route models of reading acquisition and sensory substitution

**Figure 2:**
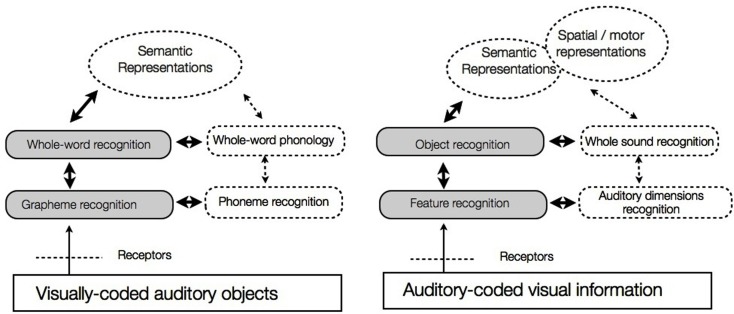
**Further analogies between reading and integration of visual-to-auditory sensory substitution devices**. Full arrows indicate new elements brought about by training and new devices or artifacts, dotted arrows indicate elements that pre-existed.

Going one step further, we want to argue that, by analogy with dual-route models of reading, learning to use a sensory substitution device is no longer to be thought of as being merely a matter of perceptual learning or adaptation, but as the building of a parallel access to cognitive and spatial representations that get grafted onto some pre-existing perceptual-cognitive route (e.g., sounds to objects and spatial representations in the case of the vOICe, see Figure 2). This analogy encompasses the existing evidence and allows further generalization or predictions which can promisingly be put to test.

First and foremost, the dual-route model of integrating sensory substitution devices is more illuminating with respect to the existing results on training. Instead of trying to decide between the cognitive or active aspects of the integration of sensory substitution devices, or to adjudicate the exact importance of an explicit teaching of rules (Auvray et al., 2007b; Siegle and Warren, 2010), the dual-route model stresses the *complementarity* between these two aspects. As in reading, a combination of practical active training and explicit teaching of codes is the most common method in the area of sensory substitution, and the one for which most of the results have been collected.

One objection here might come from the fact that explicit teaching of the coding rules is not necessary for users to start showing improved performance with a sensory substitution device. This can nonetheless be accommodated within the analogy with reading. In some cases of sensory substitution or reading, a minimal competence arises without training through the direct association of a certain novel stimulus with a referent given through another modality: as much as children can learn to associate the complex visual form of a certain word with its phonology, and/or its referent – for instance, associate the visual look of the word “car” to the sound (car) and from there to what they know about cars – novice users of auditory-to-visual devices like the vOICe can learn to associate a set of auditory patterns obtained through their headphones to an object recognized by touch (and/or labeled by the experimenter). Such an associative strategy though is unlikely to generalize and predicts that same amount of effort/time will be needed to learn every novel item. Another possibility, again opened by the comparison with reading, is that the code can be (at least to a certain extent) intuitively figured out, as it happens with young children learning to read before any formal training.

A more specific prediction, here, is that learning without explicit teaching will be more frequent or easy with visual-to-tactile devices, at least in sighted and late-blind individuals, as they rely on natural crossmodal equivalences between tactile and visual shapes which do not need to be independently taught (Spence and Deroy, 2012). By contrast, visual-to-auditory devices benefit from less immediate transfers. This, however, does not mean that their integration cannot be helped by pre-existing audio-visual correspondences, for instance, between pitch and size, pitch and brightness, or sounds and shapes. In fact, it has been shown that most individuals tend to associate higher pitched sounds with smaller or brighter visual targets from a very early age (see Marks, 1974, 1987; see Spence, 2011 for a review) and sounds of words like “takete” or “maluma” to angular and round shapes, respectively. Interestingly, these crossmodal correspondences have an influence on a variety of tasks, including speeded detection tasks and word learning. In a forced-choice task, two and a half year-old children were asked by the experimenter to match two novel words to two visual objects. The children tended to match rounder shaped objects to words containing the vowels [a] or [u] like bamu and angular shaped objects to words containing the vowels [i]or [e] like kuh-tee (see Köhler’s, 1929, original study; Maurer et al., 2006). Many other studies have subsequently demonstrated similar effects across a variety of languages (see Imai et al., 2008; Kantartzis et al., 2011; Yoshida, 2012). People also identify novel objects more rapidly when crossmodal label-object mappings follow these crossmodal correspondences than when they do not.

Within the domain of visual-to-auditory devices, there is a likely correlation between the intuitive aspect of the code and the amount of explicit training needed to achieve a reasonable level of performance. Cutting these to a single dimension, the prediction is that, in the absence of explicit teaching, the integration of devices relying on a single and robust crossmodal correspondence (for instance, only between high-pitched sounds and brightness) will be easier than integrating a device that does not rely on such intuitive correspondences. Predictions for the amount of explicit training for devices using multiple dimensions like the vOICe are harder to make even if they use intuitive correspondences, as we lack good models of how crossmodal correspondences act in combination (see Spence, 2011 for a review; see also Proulx, 2010, for an analogous claim).

### A three level model

The benefits of the analogy with reading goes further than previous claims and observations that using sensory substitution devices is, in a sense that remain quite often under-specified, a form of sensory cross-talk or rewiring (e.g., Amedi et al., 2007; Proulx, 2010) and is akin to more widespread examples of multisensory processing. Take, for instance, the recognition of shape, known as a case of crossmodal transfer (see Streri, 2012, for a review): According to most models, shape is processed first in a modal-specific way, that is as a tactile or a visual shape, before these two can be encoded in a common, amodal format, or translated into one another (see Streri, 2012 for a review). As a result, visual and tactile perception of shapes come to give access to what is considered as a single kind of information, or property, which is neither specific to vision nor to touch. At first, it can seem appropriate to think about visual-to-auditory sensory substitution in such a way, that is as building an additional access (e.g., auditory) to shape. This model, however, might overstate the similarities between the domain of sensory substitution and the domain of crossmodal transfer in a way that one is not obliged to accept, and which hide some specificities of sensory substitution.

First, the fact that one can access information about shape through audition does not necessarily mean that there is such a thing as the perception of auditory shape, like there is a perception of visual shapes or tactile shapes. It seems like a stretch to say that sensory substitution devices can change the proper objects of audition, turning the perception of sounds into the perception of shapes in space. Variations in shape/surface are reflected by variations in the resulting optic or tactile stimuli, and can be inferred on that only basis. By contrast variations in shape/surface will not result in a variation in the auditory signal, at least not one from which that variation could normally be inferred. The only way to constrain the inference is to learn an arbitrary translation from one to the other. This is then more similar to the case of reading where variations in the shapes of words or letters do not directly lead to differences in sound. What happens though is that variations in the shapes of words or letters can be *translated* into variations in sounds, and from there to meaning. The arbitrariness of the translation is sufficient to make the inference from seeing letters to auditory properties of words and meanings special explaining for instance why a change of code requires explicit teaching of new rules of inference. Hearing through the vOICe gives cues to infer properties of shapes/surfaces in a similarly code-dependent way.

Thinking about sensory substitution as a parallel to reading skills helps introducing a distinction between two sub-levels of sensory cross-talk or conversion, paralleling the inter-related levels of separate letters, and whole word recognition. When using sensory substitution devices, recognizing objects can rely on the recognition of basic features (e.g., lines, shapes), global templates (e.g., a specific sound pattern for bottles or plants), or a balance between the two. This distinction might help to explain patterns of generalization of training, and why training with new objects is facilitated when either global templates or specific features are similar to those that have been used previously (Kim and Zatorre, 2008) – a point which is left unexplained in the perceptual model.

Now whole objects or whole word recognition is also strongly linked to pre-existing semantic representations of objects: for instance, the knowledge of what plants and keys are, is used when learning to recognize them with the vOICe. Similarly, learning to identify, for instance, the words “too” and “two” is based on previous knowledge not only of the sounds but also of the semantic values of the words. The same is true in the learning of non-homophonous words, as whole word recognition, for instance, at times dominates letter-by-letter recognition, a phenomenon known as grapheme restoration. In reading acquisition and later practice, it is not unusual to observe cases for which words differing only by one letter (orthographic neighbors, like “word” and “ward,” for instance, see Williams et al., 2006) or words with a letter-inversion (“jugde” instead of “judge,” see Acha and Perea, 2008) are confused in favor of the more contextually relevant or frequent word. A prediction of the present model is that such restoration effects might be found in sensory substitution as well, with users missing or distorting the identification of low-level features due to the recognition of global templates, and relatedly, of the attribution or expectation of a certain meaning. Given this prediction, a minor feature-inversion in the auditory conversion of an object with the vOICe should remain undetected if the global object template is familiar.

Going one step further, what the model suggests is that we will learn as much concerning the integration of sensory substitution devices from their failures or limits than from their successes. Many kinds of limits have been identified in reading and help understand how this skill develops; for instance, limits on parallel processing (see Reichle et al., 2009, for a review), or breaks in transparency and in fluent processing generated by novel instances of objects or changes in the coding, susceptibility to crowding (see Whitney and Levi, 2011, for a review). Testing whether such limits also exist in the case of sensory substitution devices opens-up an interesting area of investigation. Access to distal objects remains highly constrained by the limits set up by the learned code (parallel to the failures of recognition for rotated letters). The learning of sensory substitution devices will depend on the interactions and interferences between these three steps on the new routes (i.e., semantic, whole object, and feature recognition).

In terms of results, we need to remain aware of the limits of the analogy, coming from the disparity between the objects at stake, which are linguistic in one case and material in the other. However, the model of an inter-dependent acquisition, increased automaticity and autonomy of new sensory templates exhibited in reading skills parallels the features of integration and use of sensory substitution devices. In both cases, the new patterns of invariance across sensory stimulation are helped by different levels of inter-sensory conversions. These new patterns of invariance collaborate in successful, direct recognition of the relevant information, which remains nonetheless fragile and subject to regression, to mere noise in the case of sensory substitution device users, and mere shapes in the case of (novice) readers. Within the perceptual assumption, these limits or regressions can only be said to correlate with task difficulty and users’ experience. This remains descriptive and not explanatory. By contrast, the analogy with reading helps in shaping testable predictions and in looking for specific patterns of regression and acquisition. It is for instance well known that novice readers regress to letter-by-letter reading for long or unknown words and more generally adopt coarser and more associative strategies than trained readers (see Dehaene, 2009, for a review). An interesting parallel could be made here with sensory substitution devices, looking at whether users go back to a feature-by-feature deciphering of the scene when presented with new or more complex objects or adopt a coarser global associative strategy at the beginning of the training. In other terms, such investigation should focus on the errors made by users of sensory substitution devices during their training, and try to explain why they commit more or specific types of errors in certain cases, and not in others. Again, as it is the case with reading, we might learn about the positive skills acquired by looking at errors or difficulties encountered during acquisition.

Besides the fundamental scientific interest, this investigation is likely to help with designing better devices. Another point worth mentioning here is that, if the analogy with reading holds, the design itself will be limited by the very limits of our capacities for cognitive reorganization, as witnessed in reading: access will remain limited in terms of the number of objects for users of sensory substitution devices as with graphemes for readers.

### Consequences of the reading analogy

Theoretically speaking, it might at first seem that our analogy cancels the exciting challenges raised by the widely assumed perceptual interpretation of sensory substitution, noticeably by stopping the quest for the right kind of sensory modality to ascribe them to. Other challenges are however introduced by the reading analogy.

The most straightforward one is the need to look for a better name for these devices. Conceiving of these devices as providing their users with a skill akin to reading makes the very term of “sensory substitution” irrelevant, and even terms like “compensation” might not be appropriate. This said, there might be historical and methodological reasons to keep the name sensory substitution devices, as the one on which everyone has agreed for a long time.

More fundamentally, the comparison with reading shows that learning to use a sensory substitution device should not be thought of as occurring “horizontally”: Sensory substitution devices do not have to fit within the concept of a sense even if they apparently serve similar functions (i.e., identification and localization). On the contrary, they help introducing the notion of vertical integration: they do not fit among the sensory modalities but also they *require* the existence of (some of) these modalities. Sensory substitution devices are built up from existing sensory modalities both in terms of the receptors that they use, and of the invented code they rely on. In addition, this relation is not of emergence (i.e., as if a new sense or skill appeared that was not reducible to the previous ones) but of inter-dependence or crafting (i.e., the new skill starts from an existing one, and although it becomes progressively more independent, it does not become totally detached from the initial elements). Where do these “vertically integrated” skills fit in our models of the mind? Here, we need to point at a relatively poor philosophical literature when it comes to thinking about the status of semi-perceptual, semi-cognitive skills such as reading, and their relation to more canonical forms of perception. Many philosophical questions remain open. Does reading simply consist in *seeing* letters? What is then the difference between seeing a letter and seeing a shape? If reading is an access to meaningful words, what is the difference between words and “common sensibles” for instance, shared both by audition, vision, and eventually touch? Addressing these questions for reading, we contend, is not only intrinsically important but likely to offer a parallel to build better models of sensory substitution.

Last but not least, in recent years, sensory substitution has been increasingly seen as a window onto neurological plasticity following sensory loss. It has allowed researchers to study the neurological changes in primary and secondary sensory areas in blind users after an extended training with tactile or auditory devices (Ptito et al., 2005; Amedi et al., 2007; Collignon et al., 2007; Kupers et al., 2010; Ortiz et al., 2011). Evidence obtained with sensory substitution devices is here crucial to think about sensory rehabilitation in general (Merabet and Pascual-Leone, 2010, for a review), but the present proposal suggests that it should be studied under the heading of culturally driven plasticity (Ansari, 2012) integrating top-down influences rather than naturally occurring rewiring or crossmodal transfers as the one occurring between tactile and visual shapes for instance or in developmental synesthesia (Ward and Meijer, 2010). The effort to understand sensory substitution should be tied to the new challenges of understanding the neurological changes induced by literacy or numeracy, regarding for instance the role of top-down influences, or the effects of learning on the initial levels of sensory processing.

## Conclusion

The use and integration of sensory substitution devices are investigated within a perceptual paradigm that constrains actual investigation and biases the interpretation of the results regarding training, subjective, and neurological changes. A more comprehensive perspective is needed to take into account overlooked evidence, which is offered by an analogy with the development of reading skills (see Table 1). In brief, these devices provide a powerful means to “read” the world, but not to “see” it – which incidentally questions the relevance of the very idea of “sensory substitution.”

**Table 1:** **Summary of the positive and negative evidence or problems = raised by studies on sensory substitution**.

	Behavior			Required training		Subjective changes		Neurological changes	
Evidence	New localization and identification abilities	Limits in number of objects, role of familiarity with the object	Regress or break in transparent access	Necessary	Role of practice vs. explicit rules. Patterns of generalization	Qualitative change after training	No clear modality	Neurological plasticity in V1 for trained blind users	Is it “visual” activity? Are changes limited to visual areas?
Perceptual models	+			±	±	+		±
Reading-like model	+	+	+	+	+	+	+	+	±

*The table highlights how well each model handles or is capable of handling the existing empirical evidence (+, accounted; ±, possibly accounted; blank, not accounted). It should be clear that the reading-like model does better than perceptual models*.

Learning to use sensory substitution devices must be redefined as an inter-dependent acquisition, progressive automatization, and relative autonomy of new translation templates: letters and words act as newly visually coded speech sounds; in the same way auditory templates in sensory substitution devices like the vOICe act as newly auditorily coded visual shapes and objects. New patterns of invariance across sensory stimulation are helped by different levels of inter-sensory conversions. The overall account generates new ways of investigating the integration and use of sensory substitution devices, as well as their technological future, resulting in several recommendations.

The first, and more general one is methodological. Instead of trying to improve sensory substitution devices within the perceptual assumption, we should accept that their use and integration will remain limited and study what their limits are, and where they come from. More specifically, we would suggest that these limitations are likely to vary depending on groups of participants: like for reading skills, the degree of perceptual accuracy, as well other differences in attention or cognitive competences, will isolate different groups of participants for both neurological and behavioral studies. A more discriminated view will deliver more fine grained conclusions about how various components articulate and explain the relative success of sensory substitution devices.

A second important adjustment, theoretical this time, is to realize that the study of sensory substitution devices does not shed light on perception stricto sensu. Noticeably, these studies will be of no relevance in the debates regarding the definition or individuation of the senses and will not constitute canonical examples of what it is to perceive in a certain sensory modality. Rather, sensory substitution devices open-up questions about what we have called vertical faculties: some “hybrid capacities” might be built along sensory transducers faculties by exploiting their specific outputs, and relying on the structural features of the latter, and building new crossmodal correspondences or translations between them. What sensory substitution shows therefore is not strictly sensory plasticity nor perceptual emergence or extension, but mostly culturally driven *multisensory plasticity*, that is the margin left for exploiting and redirecting the existing rules of multisensory and crossmodal interactions to build new cognitive routes between existing components.

## Conflict of Interest Statement

The authors declare that the research was conducted in the absence of any commercial or financial relationships that could be construed as a potential conflict of interest.

## Footnotes

^1^It should be mentioned that some of these rules of translation might also be more efficient because they are more “natural” than others, that is, because of existing crossmodal correspondences between for instance auditory pitch and elevation in space (see Marks, 1974, 1987; Spence and Deroy, 2012).
